# Associations Between Depression and Gastrointestinal Disorders in a Large Real-World Cohort

**DOI:** 10.7759/cureus.105261

**Published:** 2026-03-15

**Authors:** Taylor M Gong, Zachary Li, Manasa Mula, Eduardo Espiridion

**Affiliations:** 1 Psychiatry, Reading Hospital Tower Health, West Reading, USA

**Keywords:** depression, electronic health records, gut-brain axis, inflammatory bowel disease, irritable bowel syndrome

## Abstract

Background

Irritable bowel syndrome (IBS) and inflammatory bowel disease (IBD) commonly coexist with depression; however, large-scale data on this relationship remain limited. We conducted a retrospective cohort study utilizing the TriNetX database to evaluate direction-specific associations between gastrointestinal (GI) disorders and depression.

Methodology

Adults aged 18-65 years with new diagnoses of IBS, IBD, and depression were included. Cohorts were matched to controls without the index condition, and the one-year incidence of new-onset depression after IBS or IBD and new-onset IBS or IBD after depression was assessed. We also examined the one-year depression risk among IBD patients treated with sulfasalazine compared with those treated with no sulfasalazine and mesalamine.

Results

Within one year, 6.2% of IBS patients developed depression versus 3.1% of controls (absolute risk difference = 3.1 percentage points; risk ratio (RR) = 2.00, 95% confidence interval (CI) = 1.97-2.03), and 3.9% of IBD patients developed depression versus 2.8% of controls (absolute risk difference = 1.1 percentage points; RR = 1.38, 95% CI = 1.35-1.41). In patients with depression, 0.83% developed IBS versus 0.37% of controls (absolute risk difference = 0.46 percentage points; RR = 2.25, 95% CI = 2.21-2.28), and 0.24% developed IBD versus 0.16% of controls (absolute risk difference = 0.08 percentage points; RR = 1.47, 95% CI = 1.44-1.51). In IBD patients, sulfasalazine use was associated with a higher incidence of depression compared with no sulfasalazine (12.9% vs. 3.1%; RR = 4.14, 95% CI = 3.53-4.85) and mesalamine (12.8% vs. 10.6%; RR = 1.21, 95% CI = 1.09-1.34).

Conclusions

In this large retrospective cohort study, GI disorders and depression were associated with increased risk of subsequent diagnoses in separate temporal analyses. These findings support existing literature and highlight the need for further research to clarify these mechanisms.

## Introduction

The gut-brain axis (GBA) is a bidirectional pathway that allows for communication between the gastrointestinal (GI) tract and the central nervous system (CNS). Recent reviews suggest that the gut microbiome, immune mediators, and neural signaling simultaneously modulate gut function and emotional state [1-3]. Disruption of the GBA has been linked to GI disorders and psychiatric conditions, with factors such as 5-HT (serotonin) signaling and inflammation mediating these conditions [1].

Irritable bowel syndrome (IBS) is a functional GI disorder characterized by abdominal pain and altered bowel habits [4]. Meta-analyses have shown that IBS frequently coexists with psychiatric conditions and can have a substantial impact on quality of life [5]. Inflammatory bowel disease (IBD), including Crohn’s disease (CD) and ulcerative colitis (UC), is a chronic inflammatory GI disease that is also associated with psychiatric comorbidities [6]. Additionally, pharmacologic agents used in IBD, such as sulfasalazine, have been shown to alter microbial gene expression and could plausibly affect psychiatric outcomes [1].

In this study, we sought to clarify temporal relationships between these disorders in a large, real-world population. Specifically, we aimed to (1) estimate the one-year incidence of new-onset depression in patients with IBD or IBS compared to controls; (2) estimate the one-year incidence of new-onset IBS or IBD in patients with depression compared to controls; and (3) among IBD patients, compare the risk of depression between those treated with sulfasalazine and two comparison groups, namely, matched IBD patients not on sulfasalazine and matched IBD patients on mesalamine.

## Materials and methods

Study design and cohort definition

The data used in this study were collected on September 18, 2025, from the TriNetX Global Collaborative Network, which provided access to electronic medical records (diagnoses, procedures, medications, laboratory values, genomic information) from approximately 188 million patients from 157 healthcare organizations. The TriNetX platform was used to define cohorts, run propensity score matching, and estimate incidence. All statistical analyses were conducted using the TriNetX analytics platform.

This retrospective cohort study included adult patients aged 18-65 years with first-time diagnoses (index events) of IBS, IBD, or depression recorded between 2010 and 2024. Cohorts were defined by the following International Classification of Diseases, Tenth Revision (ICD-10 codes): IBS (K58), IBD (K50, K51), and depression (F32, F33). For each exposure-to-outcome analysis, the index was the first recorded diagnosis of the exposure condition. Each direction of association was evaluated using separate exposure-defined cohorts rather than within the same patient population.

Exclusion criteria

We excluded any patient with a recorded history of the outcome condition before the index date. We also excluded patients with prior medication exposures associated with the outcome condition to reduce confounding by treatment. Specifically, for analyses where depression was the outcome, we excluded individuals with any antidepressant or antipsychotic prescription before the index. For analyses where IBD was the outcome, we excluded those with prior 5-ASA prescriptions (sulfasalazine, mesalamine, or olsalazine). For IBS outcomes, we excluded those with prior use of peppermint oil or antidiarrheals (loperamide or diphenoxylate).

Time windows and propensity score matching

Time windows for analyses were defined within TriNetX. For the IBS/IBD-to-depression and depression-to-IBS/IBD analyses, outcomes were assessed at one year (1-365 days). Outcomes for sulfasalazine analyses were assessed from 30 days after exposure to 365 days, allowing a one-month period for medication exposure. Patients were followed from the index date until outcome occurrence, loss to follow-up within the network, or completion of the 365-day observation period.

Patients with the exposure condition were propensity-score matched 1:1 to control patients without the exposure, using greedy nearest-neighbor matching without replacement (TriNetX default caliper 0.10 SD) (e.g., IBS-to-depression control group could not have been previously diagnosed with IBS). Matching covariates were chosen based on clinical relevance. In the depression-to-IBS and depression-to-IBD, we matched on age and sex (additional covariates were limited by the large cohort size). For the IBS-to-depression and IBD-to-depression analyses, we matched on age, sex, race (White, Black, or Asian), and (for IBD) subtype of IBD (CD vs. UC). In the sulfasalazine analyses, covariates included age, sex, race, IBD subtype, and baseline glucocorticoid use.

Outcomes and measures

Primary outcomes were one-year (a) incident depression after IBS or IBD; (b) incident IBS or IBD after depression; and (c) incident depression after sulfasalazine exposure (compared to mesalamine and to no sulfasalazine exposure). Data collected included cohort sizes, number with outcome, mean age, sex distribution, risk differences, and risk ratios (RRs) with 95% confidence intervals (CIs). Comparisons between matched cohorts were conducted using the statistical tools available within the TriNetX platform, which calculates RRs and associated p-values using cohort comparison methods based on the chi-square test for categorical outcomes. A two-sided p-value <0.05 was considered statistically significant.

Ethical considerations

This retrospective study was exempt from informed consent. The data reviewed is a secondary analysis of existing data, does not involve intervention or interaction with human subjects, and is de-identified per the de-identification standard defined in Section §164.514(a) of the HIPAA Privacy Rule. The process by which the data is de-identified is attested to through a formal determination by a qualified expert as defined in Section §164.514(b)(1) of the HIPAA Privacy Rule. This formal determination by a qualified expert was refreshed in December 2020.

## Results

After applying the inclusion criteria, we identified 705,792 patients with IBS and 564,435 patients with IBD, as well as approximately 7 million with depression. Baseline demographics were balanced after propensity matching (mean age = ~35 years, 40% male in groups 1-4). The medication comparison cohorts (sulfasalazine vs. no sulfasalazine, and sulfasalazine vs. mesalamine) each contained roughly 5,700 matched IBD patients (mean age = ~40, ~55% male). Balance was assessed using standardized mean differences (SMDs), with SMD <0.1 considered well-balanced. Race remained modestly imbalanced after matching in some cohorts (SMD >0.1). See Table 1 for detailed baseline characteristics.

**Table 1:** Demographic information for all cohorts. Group 1 represents incident depression after IBS, group 2 represents incident depression after IBD, group 3 represents incident IBS after depression, group 4 represents incident IBD after depression, group 5 represents incident depression after sulfasalazine exposure versus no sulfasalazine exposure, and group 6 represents incident depression after sulfasalazine exposure versus mesalamine exposure. Continuous variables are presented as mean ± standard deviation (SD), and categorical variables are presented as N (%). IBS: irritable bowel syndrome; IBD: inflammatory bowel disease

	Group 1	Group 2	Group 3	Group 4	Group 5	Group 6
Age						
Mean ± SD	35.0 ± 14.5	35.1 ± 14.4	35.4 ± 14.5	35.5 ± 14.5	40.8 ± 12.8	39.2 ± 12.4
Sex						
Male	40.0%	39.6%	40.5%	40.4%	47.6%	43.4%
Female	57.2%	57.5%	56.7%	56.8%	47.9%	53.1%
Race						
White	51.0%	51.2%	50.6%	50.7%	71.0%	74.2%
Black or African American	12.9%	12.9%	12.8%	12.9%	11.0%	9.2%
Asian	4.9%	4.9%	4.9%	5.0%	3.2%	3.1%

Incident depression after IBS or IBD

Patients with baseline IBS or IBD had a higher one-year incidence of depression compared to matched controls. In patients with IBS, 6.2% developed depression, compared to 3.1% in the matched control group (absolute risk difference = 3.1 percentage points; RR = 2.00, 95% CI = 1.97-2.03). For patients with IBD, 3.9% developed depression compared to 2.8% in the matched controls (absolute risk difference = 1.1 percentage points; RR = 1.38, 95% = CI 1.35-1.41) (Table 2).

**Table 2:** Incident depression in patients with IBS or IBD results (groups 1-2). Categorical data are presented as N (%). Relative risk values are presented with 95% confidence intervals (CIs). Statistical significance was defined as p < 0.05. IBS: irritable bowel syndrome; IBD: inflammatory bowel disease

	Cohort size	Outcome, N (%)	Risk ratio	P-value
Group 1			2.00 (1.97, 2.03)	<0.001
IBS	705,792	43,577 (6.2%)		
No IBS	705,792	21,796 (3.1%)		
Group 2			1.38 (1.35, 1.41)	<0.001
IBD	564,435	21,967 (3.9%)		
No IBD	564,435	15,944 (2.8%)		

Incident IBS or IBD after depression

Analyses using depression as the exposure also showed increases in the one-year incidence of IBS and IBD compared to matched controls. In the depression cohort, 0.83% developed IBS compared to 0.37% of controls (absolute risk difference = 0.46 percentage points; RR = 2.25, 95% CI = 2.21-2.28), and 0.24% developed IBD compared to 0.16% of controls (absolute risk difference = 0.08 percentage points; RR = 1.47, 95% CI = 1.44-1.51) (Table 3).

**Table 3:** Incident IBS (group 3) or IBD (group 4) in patients with depression results. Categorical data are presented as N (%). Relative risk values are presented with 95% confidence intervals (CIs). Statistical significance was defined as p < 0.05. IBS: irritable bowel syndrome; IBD: inflammatory bowel disease

	Cohort size	Outcome, N (%)	Risk ratio	P-value
Group 3			2.25 (2.21, 2.28)	<0.001
Depression	6,695,756	55,784 (0.83%)		
No depression	6,695,756	24,822 (0.37%)		
Group 4			1.47 (1.44, 1.51)	<0.001
Depression	7,011,639	16,606 (0.24%)		
No depression	7,011,639	11,295 (0.16%)		

Depression risk following sulfasalazine use

In the medication comparison cohorts, 12.9% of IBD patients on sulfasalazine developed depression versus 3.1% of matched IBD patients not on sulfasalazine, corresponding to a fourfold higher risk of depression (RR = 4.14, 95% CI = 3.53-4.85). Sulfasalazine-treated patients also had a higher incidence of depression than mesalamine-treated patients (12.8% vs. 10.6%; absolute risk difference = 2.2 percentage points; RR = 1.21, 95% CI = 1.09-1.34) (Table 4). These data suggest a notable association between sulfasalazine use and depression risk, which we further investigate in the Discussion section.

**Table 4:** Incident depression in patients on sulfasalazine versus no sulfasalazine (control) or mesalamine results (groups 5-6). Categorical data are presented as N (%). Relative risk values are presented with 95% confidence intervals (CIs). Statistical significance was defined as p < 0.05. IBS: irritable bowel syndrome; IBD: inflammatory bowel disease

	Cohort size	Outcome, N (%)	Risk ratio	P-value
Group 5			4.14 (3.53, 4.85)	<0.001
Sulfasalazine	5,722	736 (12.9%)		
No sulfasalazine	5,722	178 (3.1%)		
Group 6			1.21 (1.09, 1.34)	<0.001
Sulfasalazine	5,721	735 (12.8%)		
Mesalamine	5,721	609 (10.6%)		

## Discussion

Our findings demonstrate significant temporal associations between GI disorders and depression when examined in separate exposure-defined cohorts. Patients with IBS and IBD had a significantly increased risk of developing new-onset depression following initial diagnoses. IBS patients had approximately double the one-year risk of developing depression compared to controls (about a twofold increase), whereas IBD patients had a modest ~1.4-fold increase. Conversely, patients with depression had a higher risk of being diagnosed with IBS or IBD compared to those without depression, although the absolute risk increases were small. As each temporal direction was evaluated in separate matched cohorts, these findings should be interpreted as associative rather than causal.

These findings are consistent with existing literature describing interactions within the GBA. Previous meta-analyses have found significantly increased risk of new-onset depression following IBS and IBD diagnosis, with studies reporting odds ratios of 2.72 for IBS [5] and 1.42 for IBD [3]; findings that are similar to our RRs of 2.00 and 1.38, respectively. Similarly, when assessing the impact of depression, previous studies reported RRs of 1.90 for new-onset IBS [7] and 1.20 for new-onset IBD [8], consistent with our RRs of 2.00 and 1.38, respectively.

Several biological mechanisms may contribute to this association. Chronic systemic inflammation in IBD is thought to increase gut permeability and promote neuroinflammation [9], potentially linking IBD to depression. Additionally, gut dysbiosis, common in both IBD and IBS, may alter enteric serotonin production, a key modulator of mood [10,11]. The mechanism behind depression’s association with IBD and IBS is less clear, but animal models suggest that stress and depression-like states increase susceptibility to intestinal inflammation [12]. Emerging genetic studies also indicate shared loci between GI disorders and depression [13]; however, causality remains unexplored.

In addition to the findings above, we also identified sulfasalazine as a possible contributor to this relationship. When compared to patients not on sulfasalazine, sulfasalazine use was associated with a more than fourfold higher risk of depression. Furthermore, when compared to mesalamine use, sulfasalazine continued to show an approximately 1.21-fold higher risk compared to mesalamine. This marked elevation in risk exceeds what might be expected from baseline IBD-related depression rates alone, suggesting a potential drug-specific effect or confounding by indication.

Two possible explanations may account for these results. One hypothesis is that sulfasalazine’s known impact on gut microbial gene expression and folate metabolism could adversely affect mood pathways [1]. Rare case reports of sulfasalazine-induced depression have been reported in the literature [14], though no large studies have evaluated its direct impact on neurotransmitter systems. Alternatively, this finding could be due to confounding by indication or disease severity. Current guidelines reserve sulfasalazine for patients who do not respond to first-line mesalamine [15]. Therefore, the sulfasalazine cohort may represent patients with more severe IBD, a population that is inherently at higher risk of depression. We attempted to control for some markers of severity (e.g., glucocorticoid use), but residual confounding may remain.

Key limitations of this study result from the retrospective design. As cohorts were defined by ICD-10 codes, misclassification by clinicians is a possibility. We attempted to limit this by requiring first-time diagnoses and exclusion of patients with a history of outcome conditions or related medications. However, as TriNetX diagnoses are based on the first recorded codes, true disease onset may have preceded clinician documentation. Residual confounding is another limitation. Although we performed propensity score matching to limit this, we could not include all factors that may have led to unmeasured differences between cohorts (e.g., socioeconomic status, lifestyle factors, and medication adherence). Additionally, residual imbalance in race may have contributed to residual confounding, and differences in healthcare utilization may have increased diagnostic coding, leading to increased identification in certain cohorts. Another limitation is that not all relevant confounders could be matched for. The depression cohort’s large sample meant we could only match for age and sex in these analyses, allowing for potential confounding to occur. Lastly, analyses were conducted within the TriNetX platform, which limited statistical testing to the methods available within the system and limited the ability to perform customized statistical modeling or additional sensitivity testing. Despite these limitations, the large sample size and real-world data provide findings that complement existing literature.

## Conclusions

In this large retrospective cohort study, IBS and IBD were associated with an increased risk of subsequent depression, and depression was similarly associated with an increased risk of subsequent GI diagnoses in separate temporal analyses. These findings support the presence of clinically meaningful associations between GI and psychiatric conditions, though causality cannot be established. Among patients with IBD, sulfasalazine use was also associated with a higher incidence of depression compared with other treatment groups, a finding that warrants further investigation. Future studies incorporating disease severity, treatment response, and longitudinal follow-up are needed to explore the mechanisms underlying these associations.
